# The Bones of the Leg

**Published:** 1888-09-29

**Authors:** 


### Physiology and its Uses.

### THE BONES OF THE LEG.

The bones of the leg are generally similar to those of the arm, and the bones of the foot to those of the hand. If Darwin's judgment be conclusive the progenitors of man were originally quadrupeds, and during the four-footed period there was probably a greater degree of similarity between the fore and hind limbs than we see now between the upper and the lower. At present there is nothing either of the quadrupedal or the quadrumanal about man. He is clearly bipedal and bimanous in the most pronounced degree. To quote an eminent anatomist: "The body of man, unlike that of animals, is, for the purpose of station or progression, balanced on one or both pelvic limbs, which are extended to a straight line at the knee-joint. The lower limb is remarkable for its length and strength. The foot of man alone" (and this is a significant circumstance) "has an arched instep, and it likewise presents a great breadth of sole. The great toe is distinguished by its large development, and, especially from that of the quadrumana, by its want of opposability, being formed, not for grasping, but for supporting the weight of the body and giving spring to the step." There is thus in this, as in other fundamental points, the most marked difference between man and the dog or horse on the one hand, and between man and the monkeys on the other. That man and the monkeys and dogs are constructed on a similar general plan is as evident as that the gooseberry bush and the oak both belong to the wooded order of vegetation. It is, however, equally evident that the ape or the dog shows no more likelihood of overtaking man in the race of development than the gooseberry manifests of catching up the oak. Purpose, order, limitation, law, are everywhere present and everywhere permanent. To deny this is to be wilfully or stupidly blind.

It was shown in a recent paper that the shoulder-blade and the collar-bone constituted together the first stage of the upper limb. Similarly half the pelvis, which in the embryo stage consists of three separate bones, is the commencement of the lower. The two halves of the pelvis unite to form a basis for the trunk on the one hand and for the leg on the other. A portion of the pelvis finds its homologue in the shoulder-blade. The hip-joint, one of the most important joints in the body for many reasons, is a "ball and socket" or "cup and ball" joint. The "cup," which is a very deep one, is formed by the pelvis, and the ball is the head of the femur. Unlike the shoulder-joint, it is one of the most difficult joints in the body to dislocate; and that is a very fortunate circumstance, for it is equally difficult to reduce. Many a "bad quarter of an hour" is spent by the second years' medical student in his endeavours to form an intelligent comprehension of the best way of reducing a dislocated hip.

The first bone of the leg proper is the femur. It is a truly noble part of the skeleton. Whether we regard its clean and handsome appearance, its shapely form, its evident adaptability to its functions, or its value as a weapon of defence in time of need, it is bound to excite our sincerest admiration. The head is like the half of a round ball, and is supported on a long, but wonderfully strong, neck. Below this, again, is a pair of splendid shoulders called *trochanters*; and then follows the trunk or shaft At the lower end the bone expands into two "condyles," which meet a similar expansion of the tibia or shin-bone, and with it form the knee-joint, the largest joint in the body. The two femurs may be regarded as two pillars, which, springing from the knee-joint, support the whole weight of the body. At the head and neck they are fastened by firm but mobile ligaments to the pelvis. Huge, fleshy, muscular masses pass from the pelvis to the femur, which give the hip-joint its various and powerful movements. Other muscles pass from the pelvis to the knee-joint and help its functions. The hip-joint is liable to disease of the most painful, enduring, and often fatal character. At the very outset of any pain in the hip or in the knee medical aid is to be sought with the utmost promptitude. Hip disease treated skilfully and at once very often results in a satisfactory cure; but if neglected for a considerable time leads to permanent disfigurement and disability, and not seldom to death.

The femur is not easily broken. When it is, the accident is frequently of the most serious kind. It may be fractured in different places, as in the neck, the lower third, and so on. A fracture of the neck is a most alarming accident, and, on account of its position, exceedingly difficult to treat. In old persons fractures of the femur are not uncommon, because of the great brittleness of the bones in the aged. In many cases a fracture of the femur in an old man or woman is the beginning of the end. The opposing ends of the bone seldom unite, and the patient, compelled to remain in bed and in one posture, generally soon dies. Whilst considering the femur it will be opportune to mention the very large thick nerve which lies behind and to the outer aspect of it. This is the great sciatic nerve. It is very large, gives off many branches, and, being the principal nerve concerned in walking, does a principal share of the work of the body. On account of its size, its branches, and its work, it requires a great deal of nutriment for its health and comfort; and when the system is feeble, and poorly supplied with blood, it is generally one of the first nerves in the body to feel its deprivation, just as a big, hungry man will sooner feel the pinch of "short commons" than a small and less craving one. This is a fact worthy of being borne in mind for a practical purpose. When the huge nerve cries out with pain (the pain of sciatica), its owner should say to himself, "My health is low. I must seek rest, or more food, or physic, or something. At any rate, I had better consult the doctor, and do what he thinks best." Many a man who has despised the warnings of repeated sciatica has paid very dearly for his folly.

The knee-joint, which, as has been said, is formed by the expansion into "condyles" of the lower end of the femur and by a similar expansion of the upper end of the tibia, or shin-bone, is not only one of the largest but also one of the most important joints in the body. Immediately in front of it is the knee-pan, or patella, often called the "knee-cap." The patella is an example of a sesamoid bone, that is, a bone not formed in the usual way as a necessary part of the skeleton, but formed, so to speak, in the course of the tendon. It protects the knee, and also, which is more important, gives attachment to muscles, and acts variously as a fixed point for muscular effort. It may be stated generally that the knee-joint is a kind of "glass house" in the bodily economy. It is so large, so constantly in use, so freely exposed to all kinds of casualties, that it probably suffers more from the general circumstances of its environment than any other joint. Not only is it liable to a great variety of accidents and diseases, but the diseases and injuries themselves are often extremely painful and dangerous to health and life. It is impossible to describe these various diseases and accidents here, but the warning so often given about calling in prompt medical attendance may be repeated with double emphasis with regard to injuries and diseases of the knee as well as of the hip.

The *tibia*, or shin, is a fine, sturdy-looking bone, second only to the femur in size. It has by its side a small companion—the *fibula*. Most people know what it is to "bark the shins." The reason why the shin is so easily "barked" and why the pain is so intense is that the front and inner aspect of the tibia is subcutaneous. There is no fleshy mass of muscle under the skin, as there is in the thigh or the calf. A kick on the calf is nothing; a kick on the shin makes the most experienced football-player wish to use both tongue and fists in reply. At the back of the tibia and fibula are the muscles of the calf. These pass down, and several of them unite to form the large tendon which lifts up the heel in walking. This is the tendon Achilles, an exceedingly light and strong band. It is sometimes injured in climbing hills and dancing, and the injury is of a very disabling character. The tibia on the inner side of the leg and the fibula on the outer make the two sides of the ankle-joint, and into the space so formed the top of the foot is fitted. The ankle-joint is a kind of hinge, and allows of movement very freely in upward and downward directions. There is a slight power of lateral and rotatory movement as well, but the free rotation of the foot can only be effected in company with the leg. For variety and freedom of movement all the joints of the leg are wonders of successful mechanism. As may be expected, fractures of the tibia, or thick bone of the lower leg, are less common than those of the fibula. The latter is what is called the "small" bone of the leg, and is often broken, especially near the lower end. This accident, when the fracture is "simple," is not very serious, though it always demands prompt treatment at the hands of a skilled practitioner.

The bones of the foot resemble generally the bones of the hand, with, however, decided differences necessitated by function. There is a *tarsus* corresponding to the *carpus* of the hand, and consisting of seven bones; a *metatarsus*, answering to the *metarcarpus* of the hand, and having the same number of bones, viz., five. The toes have their homologues in the fingers, the great toe being brother to the thumb, and having, like it, two phalanges. The other toes, like the fingers, have three phalanges each. The foot, with its toes, is for "station and progression," not for grasping and tool using. The feet of certain monkeys are hands as well as feet, but also their hands are feet as well as hands. These are the quadrumanous or four-handed monkeys. In some human beings there appears a tendency to revert to the four-handed condition. They can use their toes for grasping, writing, and other purposes in a very remarkable manner.

Although the bones of man may be considered fixed and definite as to their number, shape, size, and function, and are usually obedient to the general plan of their construction, there seems to be no doubt that modifications of a very decided kind may take place. But though this is so, there is no evidence that the modifications are of such a kind and degree as to amount to a "substitution" either in general plan or in important detail. Darwin relates, on apparently good authority, that "the Quechua Indians, who inhabit the lofty plains of Peru, have, from continually breathing a highly rarified atmosphere, acquired chests and lungs of extraordinary dimensions." There must have been in these cases a corresponding growth of the ribs. The Aymaras, an allied race who live at a height of between 10,000 and 15,000 feet, differ conspicuously from the normal type of man in the length and circumference of their bodies. Strange to say there is no corresponding increase of other parts, or we might have expected the gradual development of a race of giants at these elevations. On the contrary, as if to keep them within the limits of size proper to man, their arms are shorter than those of Europeans, and much shorter than those of negroes. Their legs, too, are shorter. They present also this peculiarity, that the thigh bone or femur is shorter than the tibia or large bone of the lower leg. Darwin suggests, and in this he is agreed with by other naturalists, that the shortening of the thigh bone, that is of the bone nearest the body, is a case of natural compensation for the elongation of the trunk. From the point of view of the student reading for examination the "bones" are an "unmitigated nuisance." From the standpoint of the man of science, they may, on account of their size and power of resisting decomposition and retaining their shape after hundreds and in some cases thousands of years, be regarded as the most important parts of the human, or animal organism.

